# Research on Impact Resistance of Reinforced Concrete Beams Strengthened with Carbon Fiber Reinforced Polymer Grid and Engineered Cementitious Composites

**DOI:** 10.3390/polym14101951

**Published:** 2022-05-11

**Authors:** Zhihao Si, Fan Liu, Jianwu Pan, Hao Dong

**Affiliations:** Department of Civil and Airport Engineering, Nanjing University of Aeronautics & Astronautics, Nanjing 211106, China; sizhihao@nuaa.edu.cn (Z.S.); lf19852830290@126.com (F.L.); nuaadonghao@nuaa.edu.cn (H.D.)

**Keywords:** impact, reinforced concrete beams, strengthen, carbon fiber reinforced polymer, engineered cementitious composites

## Abstract

When reinforced concrete structures are subjected to impact loads, they may suddenly yield or fail, or even collapse as a whole. In this paper, the impact resistance of reinforced concrete (RC) beams strengthened with carbon fiber reinforced polymer (CFRP) grid and engineered cementitious composites (ECC) was studied. Drop hammer impact tests were conducted on eight beams, then the finite element model was used to simulate the impact test, finally a simplified two-degree-of-freedom (TDOF) model was proposed for CFRP grid reinforced ECC layer strengthened RC beams under impact loading. The results showed that CFRP grid reinforced ECC layer significantly improved the impact resistance of RC beams. When the ECC and CFRP grid were used, the crack development was inhibited after the concrete cracked in the tensile area, avoiding the brittle damage of concrete beams with one crack to the end. Compared with the control beam, the reaction force of RC beams strengthened with CFRP grid and ECC under impact load increased by 16.2%~34.5%, the maximum mid-span displacement decreased by 16.3%~31.6% and the mid-span residual displacement decreased by 36.02%~49.53%. The finite element model and the proposed TDOF mode were demonstrated to effectively simulate the strengthened beam under impact loading.

## 1. Introduction

Reinforced concrete structures are threatened by many impact loads. On the one hand, the impact load comes from the destruction caused by explosion attack and ship collision with bridge; on the other hand, it comes from natural disasters such as earthquake and tsunami. When a reinforced concrete structure is subjected to impact loading, it may suddenly yield or fail, or even collapse as a whole. As the basic component of reinforced concrete structure, it is necessary to study the strengthening of reinforced concrete beam under impact load.

At present, the use of FRP for reinforcement and strengthening of concrete structures [1,2,3,4,5] is more and more extensive, among the more recent methods are FRP bar strengthened ECC [6,7,8,9] and FRP sheet strengthened ECC [10,11,12], etc. The strengthening methods for RC beams under impact loading are mainly strengthened with FRP sheet/plate [13,14,15,16,17], fiber reinforced concrete [18,19], externally bonded steel plate [20,21], high strength steel wire mesh and high-performance mortar [22,23], etc. Although these methods can improve the impact resistance of beams, they are still prone to debonding/peeling damage, poor crack control and difficult construction.

In recent years, some novel methods are presented, such as composite reinforcing system and prefabricated FRP composite repair system [24,25]. They reveal the high potential of FRP to provide safe and reliable systems. The FRP grid reinforced ECC strengthening method is also a technology with great application potential. FRP grid reinforced ECC strengthened beam has good bending resistance. CFRP grid has higher utilization rate of tensile strain than CFRP sheet, which is more advantageous in bearing capacity. CFRP grid is more secure than CFRP sheet, and has more obvious prognosis before debonding. ECC multi-point cracking feature makes the strengthened beam have more deformation ability and smaller crack width [26]. The greater the thickness of BFRP grid, and the longer the bonded length of strengthening layer are used, the more obvious the improvement of yield load and ultimate load of strengthening beam is observed. The longer the strengthening layer is used, the smaller the maximum shear stress at its end is observed, which is much smaller than its ultimate shear stress [27].

FRP grid reinforced ECC strengthened beam has good shear performance, it has better load bearing capacity, ductility and damage tolerance [28]. BFRP grid reinforced ECC strengthening layer can reduce the strain of hoop reinforcement, and the greater the thickness of the grid, the more obvious the strain reduction of hoop reinforcement, and this method can improve the bearing capacity and ductility and has stronger adhesion with concrete than BFRP sheet [29]. The increase of the reinforcement ratio of the BFRP grid and the decrease of the shear span ratio of the beam are beneficial to improve the shear performance of the beam. The failure of the BFRP grid reinforced ECC strengthening layer and the concrete occurs in the shear span area of the concrete instead of at the interface, which indicates that the strengthening layer has good adhesion with the concrete and effectively improves the shear performance of the beam [30]. The greater the amount of CFRP grid per unit length is used, the more obvious the shear bearing capacity improvement is observed. The damage mode of CFRP grid reinforced ECC strengthened beam is mainly debonding/peeling damage, and there are forms of concrete crushing damage in the shear compression zone or support zone [31].

FRP grid reinforced ECC strengthening method can improve the bonding properties of the structure. ECC as the matrix of CFRP grid cannot completely suppress the debonding caused by mid-span cracking, but compared with epoxy mortar, the characteristics of multi-point cracking make the interface shear stress transfer more uniform and better ductility. Prefabricated CFRP grid reinforced ECC layer combined with epoxy resin adhesive strengthening can suppress the debonding caused by end debonding and mid-span cracking without additional anchoring measures [32].

According to the above literature review, although there have been some studies on FRP grid reinforced ECC strengthened beams, there are few studies on the impact performance of strengthened beams. In addition, some specific issues concerned in engineering applications still lack systematic research, such as the reasonable fiber content of ECC, the reasonable thickness of ECC, and the reasonable number of layers of FRP grids, etc.

For these reasons, in this paper, CFRP grid reinforced ECC strengthening technology was used. ECC has good crack control ability and FRP is lightweight, high strength and durable. However, the traditional FRP sheet is prone to peel damage, and its binder is mostly epoxy resin that is less durable. CFRP has more advantages in terms of specific strength and modulus, and the form of grid is not easy to peel off. The fibers in ECC were selected from polyethylene (PE) fibers, which have the highest specific strength and specific modulus of elasticity among commercially available fibers in the market and are less costly.

In this paper we attempted to carry out systematic research on the influence of various parameters concerned in engineering applications. Since the FRP grid reinforced ECC strengthening method could be regarded as an improvement of the FRP sheet strengthening method, a comparison between FRP sheet and FRP grid was considered in this test. According to the experience related to the traditional FRP sheet strengthening method and the steel plate strengthening method, the strengthening effect might be not good when the number of layers and thickness of FRP sheet and steel plate were too large. Therefore, in this paper we also varied the number of layers of FRP grids and the thickness of ECC to observe the effect of these parameters on the strengthening effect. In addition, the fiber content of ECC had a significant effect on the performance of ECC, then its effect on the strengthened beam was also worth studying. The effect of different impact heights was also considered in this test.

In this paper, the effects of CFRP grid layer number, ECC thickness, fiber content and CFRP morphology on the impact resistance of strengthened beams were systematically studied. Impact tests on eight beams were conducted to investigate the effects of different conditions on their impact resistance. The tests were also numerically analyzed using LS-dyna and parametric analysis of CFRP grid thickness and longitudinal tensile steel reinforcement ratio was carried out. A simplified two-degree-of-freedom (TDOF) model for CFRP grid reinforced ECC strengthened RC beams under impact loading was proposed, and parametric analysis of shear span ratio, drop hammer impact velocity and drop hammer quality were carried out.

## 2. Experimental Content

### 2.1. Material Preparation

ECC was made by mixing cement, quartz sand, coal fly ash, water, water reducing agent, methylcellulose and PE fiber, and the mixing ratio (mass ratio) was cement: quartz sand: fly ash: water: water reducing Agent: methylcellulose = 1:0.37:0.33:0.31:0.02:0.0007, and the volume fraction of fiber content was 2%. The cement was P.O52.5 ordinary silicate cement, which was produced by Jiangnan Onoda Cement Company, Nanjing, China. Quartz sand was produced by Nanjing Daoqin Building Material Company, Nanjing, China with particle size range 80–120 mesh. The fly ash was Class I fly ash produced by Segjiang Concrete Company, Liyang, China. The fiber was produced by Hunan Zhongtai Special Equipment Company, Changde, China with length 12 mm and density 0.97 g/cm^3^. The water reducing agent was poly carboxylic acid high performance water reducing agent with 35% water reduction rate, which was produced by Jiangsu Subot New Material Company, Nanjing, China. Methyl cellulose was produced by Tianjin Damao Chemical Reagent Factory, Tianjin, China, which increased the viscosity of the slurry and was conducive to uniform fiber dispersion.

### 2.2. Material Properties

The 28-day compressive strength of the concrete was 20.3 MPa. The rebar were tested by 300 kN electronic universal testing machine for tensile testing, and the material performance indexes of rebar are shown in Table 1. According to “Standard for Test Method of Performance on Building Mortar” (JGJ/T70-2009), ECC compressive specimen was 70.7 mm × 70.7 mm × 70.7 mm prismatic specimen, and ECC flexural specimen was 40 mm × 40 mm × 160 mm prismatic specimen. According to the Japan Civil Engineering Association specification [33], the ECC tensile specimen was in the shape of a dog bone. 1000 kN compression testing machine was used for cubic compressive test of ECC, and testing antifracture machine for prismatic flexural test and 20 kN electronic universal testing machine for dog bone tensile test. The material performance indexes of ECC are shown in Table 2. Carbon fiber sheet (to compare) was CFS-1-300 unidirectional carbon fiber sheet with density of 1800 kg/m^3^ from Carbon Composites Co, Tianjin, China. The material performance indexes of carbon fiber sheet are shown in Table 3. The FRP grid was CFN L500 (unidirectional CFRP grid) from Carbon Composites Co, Tianjin, China. The transversal direction of the grid was carbon fiber with 2.5 cm spacing, with a density of 1800 kg/m^3^, and a single width of 4 mm. The longitudinal direction of the grid was glass fiber (GFRP) with 2 cm spacing and a density of 2400 kg/m^3^ and a single width of 3 mm. The material performance indexes of FRP grid are shown in Table 4.

### 2.3. Design of Specimen

**Figure 1:**
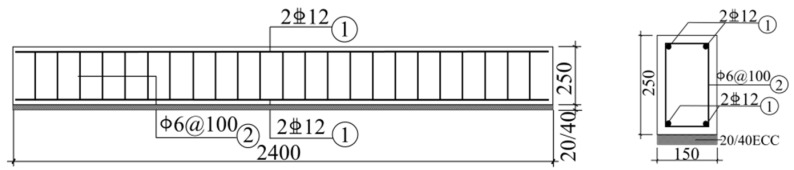
Beam reinforcement and section size.

The sectional dimensions of a total of eight RC beams was b × h =150 mm × 250 mm. The beam length was 2400 mm. The net span length was 2000 mm. Beam tension zone longitudinal reinforcement and compression zone erection reinforcement were symmetrically reinforced. Two 12 mm diameter HRB400 ribbed rebar were used as tensile longitudinal reinforcement and 6 mm diameter HPB300 plain round rebar were used for hoops with a spacing of 100 mm. The reinforcement and sectional dimensions are shown in Figure 1, and the beam parameters are shown in Table 5. L1 was a plain reinforced concrete beam. L2 and L3 were used to study the effect of the existence or nonexistence of ECC on the impact resistance of beams. L4 and L3 were used to study the effect of CFRP morphology (grid and sheet). L5 and L3 were used to study the effect of the number of layers of CFRP grid. L6 and L3 were used to study the effect of ECC thickness. L7 and L3 were used to study the effect of impact height. L8 and L3 were used to study the effect of PE fiber content. There was only one sample per specimen.

### 2.4. Test Equipment and Measurement Scheme

**Figure 2:**
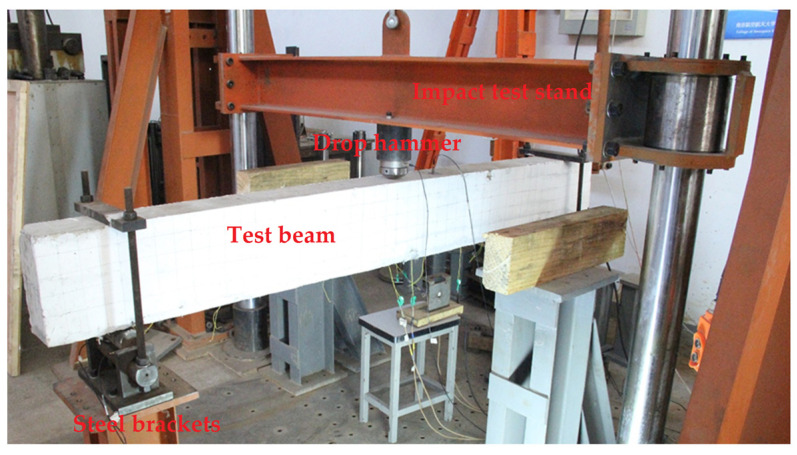
Physical diagram of impact test.

Since there was no Chinese standard for low speed impact test method of concrete specimens, the test design and the test procedure in this paper referred to the test methods in the relevant literature [34]. The drop hammer test frame used for the test is shown in Figure 2. The weight of the falling hammer was 200 kg and the maximum test height was 1 m. The test was designed with a fixture to release the concentrated force generated by the impact at both ends of the restraint. The fixture consisted of steel plate, long threaded rod, semi-cylinder and base.

**Figure 3:**
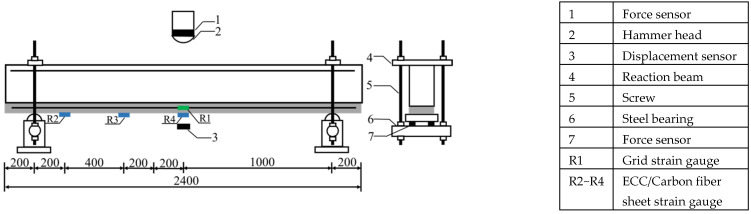
Schematic diagram of impact test.

The measurement scheme is shown in Figure 3. A force sensor with a range of 60 t was placed above the hammer head to collect the impact force. Two force sensors with a range of 10 t were arranged on each side of the support ends to collect the support reaction force. A laser displacement sensor with a range of 120 mm was arranged in the bottom span of the beam to collect the mid-span deflection. A 10aa strain gauge was attached to each of the bottom mid-span, 40 cm from the middle of the span and 80 cm from the middle of the span to measure the ECC or carbon fiber sheet strain (BX120-10AA can be used to test the strain of composite materials. The self-contained conductor and the long conductor for testing are connected by crimping, which does not require any welding during the test, and can be used with any static and dynamic strain gauges such as YSV8316 and YSV7008). Two 10aa strain gauges were applied at the middle of each grid layer to measure the strain of the CFRP grid.

Before the formal experiment, a hammer was used to gently strike the support position at the top of the beam and whether the acquisition instrument indicates a change was observed, from then on to check whether the sensor works properly. After the check, the decouple was installed above the hammer and the hammer was raised to the required height for the experiment. When the test was formally conducted, the hammer was released, then the impact force and acceleration and other data were got from each sensor, and the test process were recorded with a motion camera.

## 3. Analysis of Experimental Results

### 3.1. Crack Development

The experiment was recorded using a motion camera to recorded the crack development process at 500 fps, with the images accurate to 2 ms. Only representative L1, L2, L3 and L4 beam cracks development processes were given, the rest of the beams’ cracks were similar to L3. The development of cracks in the test beams are shown in Table 6.

#### 3.1.1. Crack Development in L1 Beam

**Figure 4:**
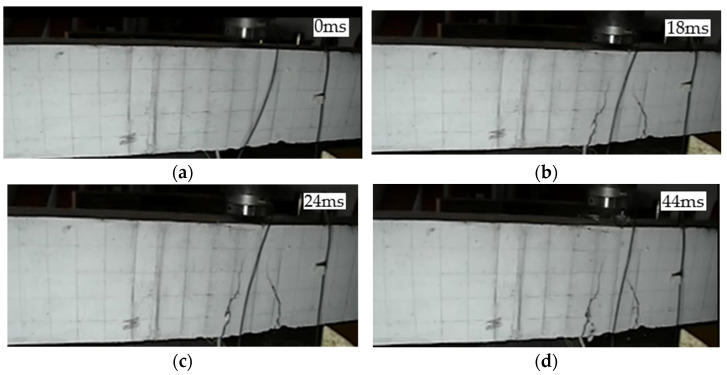
Crack development process of beam L1: (**a**) 0 ms; (**b**) 18 ms; (**c**) 24 ms; (**d**) 44 ms.

L1 beam was an unstrengthened beam. The crack development is shown in Figure 4. At 18 ms, transverse cracks appeared at the top of the beam in the hammerhead impact area, and the cracks continued to extend upwards to four-fifths of the height of the beam. At 24 ms, the mid-span displacement of the beam reached the maximum value, and the concrete in the impact area at the top of the beam had been crushed, then the hammer head and the beam rebounded upward together. At 44 ms, the hammer head broke away from the surface of the beam.

#### 3.1.2. Crack Development in L2 Beam

**Figure 5:**
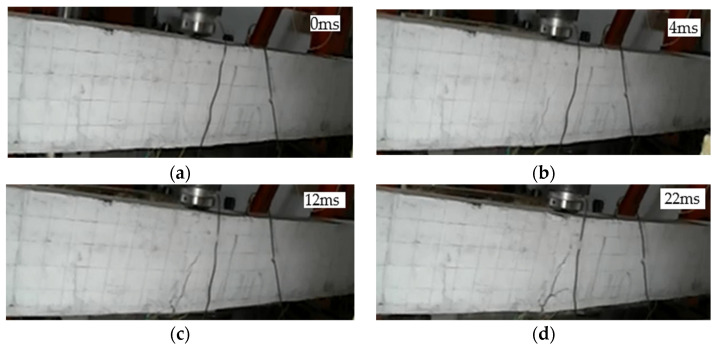
Crack development process of beam L2: (**a**) 0 ms; (**b**) 4 ms; (**c**) 12 ms; (**d**) 22 ms.

L2 was a strengthened beam with ECC layer only, and the drop height of the hammer was 1 m. The crack development is shown in Figure 5. At 4 ms, a crack appeared at the bottom mid-span, and the apex of the crack reached one-half of the height of the beam. At 12 ms, the crack continued to extend upward to three-fifths of the height of the beam, and transverse cracks appeared in the impact area at the top of the beam. At 22 ms, the mid-span displacement of the beam reached its maximum, the apex of the crack reached seven-tenths of the height of the beam, and the concrete in the impact area at the top of the beam was crushed. At 42 ms, the hammer head started to break away from the beam surface and the beam started to fall back.

#### 3.1.3. Crack Development in L3 Beam

**Figure 6:**
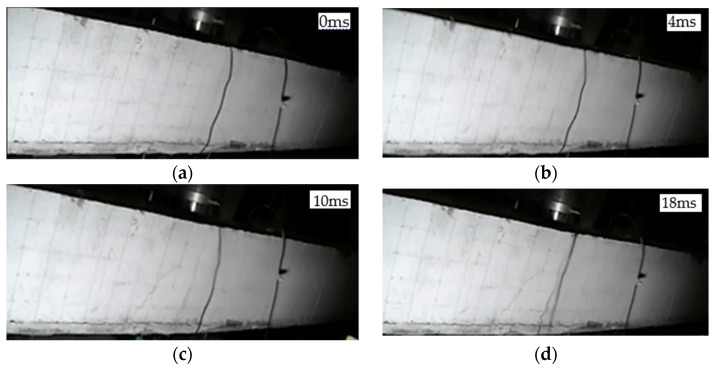
Crack development process of beam L3: (**a**) 0 ms; (**b**) 4 ms; (**c**) 10 ms; (**d**) 18 ms.

L3 was a CFRP grid reinforced ECC strengthened beam with a drop hammer drop height of 1 m. The crack development is shown in Figure 6. At 4 ms, a vertical crack appeared at the bottom of the span. At 10 ms, the crack extended rapidly to seven-tenths of the height of the beam, and a new diagonal crack was produced. At 18 ms, the span displacement of the beam reached its maximum, and the apex of the main crack reached four-fifths of the height of the beam at this time. At 40 ms, the hammer head was detached from the surface of the beam, and the beam began to fall back.

#### 3.1.4. Crack Development in L4 Beam

**Figure 7:**
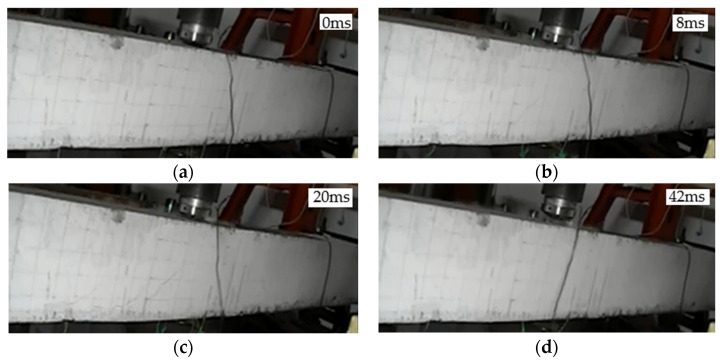
Crack development process of beam L4: (**a**) 0 ms; (**b**) 8 ms; (**c**) 20 ms; (**d**) 42 ms.

L4 was a carbon fiber fabric strengthened ECC strengthened beam with a drop hammer drop height of 1 m. The crack development is shown in Figure 7. At 8 ms, small cracks appeared at the bottom 15 cm from the mid-span, with the apex reached one-half the height of the beam. At 20 ms, the displacement in the mid-span reached the maximum, and the maximum crack apex reached three-fifths of the height of the beam. At 42 ms, the hammer head was separated from the beam surface and the beam started to fall.

### 3.2. Damage Pattern

**Figure 8:**
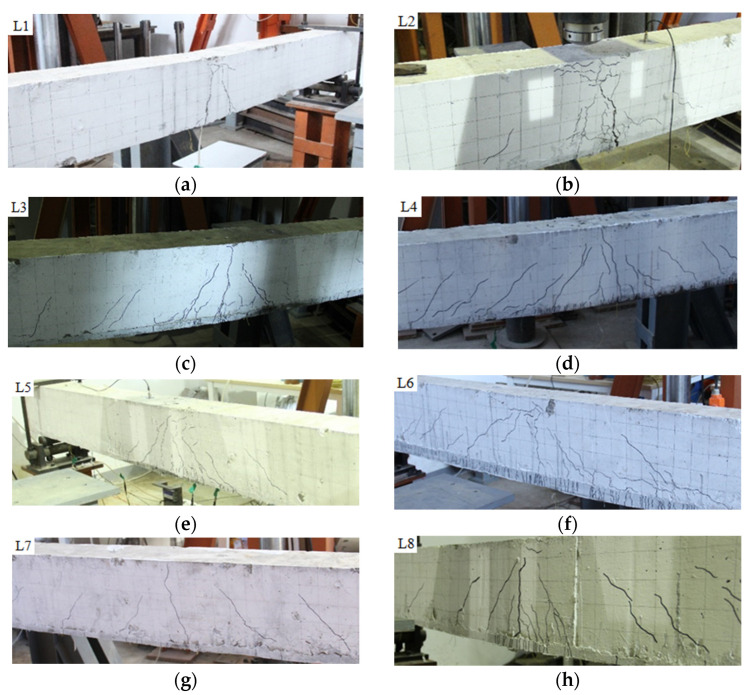
Final crack profiles of specimens: (**a**) L1; (**b**) L2; (**c**) L3; (**d**) L4; (**e**) L5; (**f**) L6; (**g**) L7; (**h**) L8.

**Figure 9:**
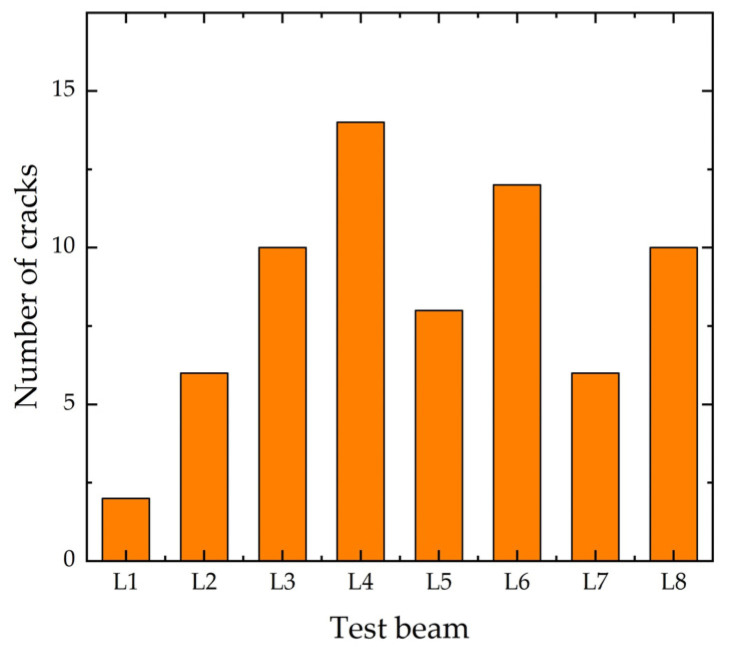
Final number of cracks.

The final crack profiles of the eight beams under impact loading are shown in Figure 8, and the final number of cracks is shown in Figure 9. The unstrengthened beam L1 had two main cracks symmetrically distributed along the bottom of the beam in the direction of approximately 75°. The number of cracks in the strengthened beam L2 with ECC had slightly increased, except for the main cracks developed vertically in the middle of the span, a few symmetrical diagonal cracks in the direction of about 45° were distributed on both sides, and a few fine cracks appeared in the ECC at the bottom of the beam. The number of cracks in beams L3, L5, L6 and L8 strengthened with CFRP grid reinforced ECC increased significantly, and the distribution patterns were similar: the width of cracks in the middle of the span was the largest, there were more diagonal cracks on both sides of the mid-span, but the width was smaller, and more vertical parallel fine cracks appeared in the ECC at the bottom of the beam. CFRP grid and ECC increased the fine cracks and shortened the length of the main cracks in RC beams under impact loading. Increasing both the thickness of ECC and the number of grid layers reduced the number of cracks, and the higher the PE content in ECC, the lower the number of cracks. The number of cracks in L7 at 0.5 m impact was much less than the case of 1 m, but the crack distribution was similar. The number of cracks in L4 was closed to that of CFRP grid reinforced ECC strengthened beam. Overall, the cracks in the strengthened beams mainly developed in the middle of the span, with smaller diagonal crack widths on both sides of the span, and the damage pattern was the development of cracks upward to concrete crushing area in the middle of the span, reflecting the characteristics of bending failure. For the strengthened beam with ECC and CFRP grid, the crack development was inhibited after the concrete cracking in the tensioned area, and the tensile force was transmitted to the uncracked concrete area, avoiding the brittle damage of concrete beams that completely crack from the bottom to the top.

### 3.3. Support Reaction and Mid-Span Displacement

#### 3.3.1. Time-History Curve of Support Reaction

**Figure 10:**
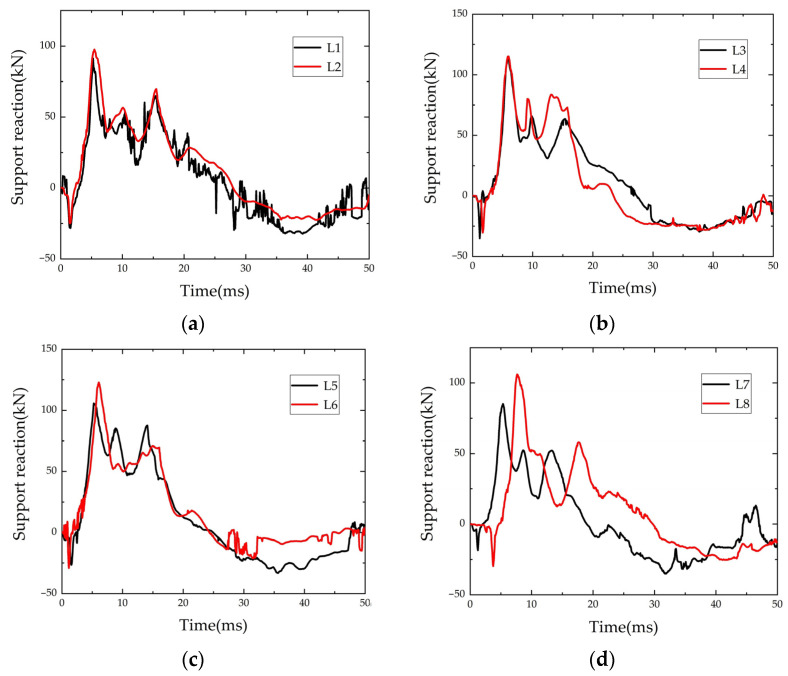
Time-history of reaction force: (**a**) L1 and L2; (**b**) L3 and L4; (**c**) L5 and L6; (**d**) L7 and L8.

The magnitude of the support reaction force obtained from the test was the sum of the measured values of the four sensors. The time-history curves of the support reaction force of each specimen are shown in Figure 10.

As can be seen from Figure 10, after the falling hammer contacted the beam, the support reaction force appeared to increase negatively, and then rapidly increased positively after reaching the negative peak, until the positive peak. The negative value was generated because the fixture used to fix the beam had pre-pressure on the force transducer before the test starts, and the specimen tended to move upward due to inertia under the impact, separating from the force transducer and releasing the pre-pressure. Kishi [35] considered that the support reaction force could evaluate the impact resistance of the beam. Therefore, the improved degree of strengthening effect of the beam could be reflected by the elevation of the bearing reaction force of the strengthened beam measured in the test.

#### 3.3.2. Time-History Curve of Mid-Span Displacement

**Figure 11:**
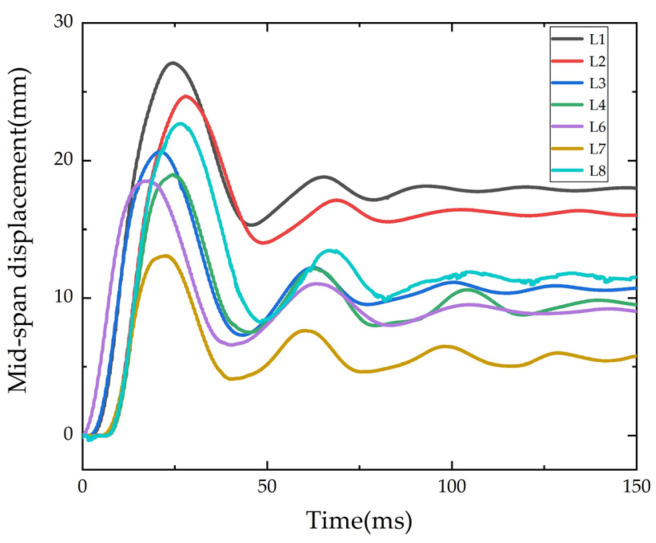
Time-history of mid-span displacement.

The displacement time-history curve of the beam under the impact load is shown in Figure 11. The mid-span displacement of the specimen increased rapidly to the peak under the impact, then sprang back under the recovery force, and the mid-span displacement decreased, so repeatedly, the vibration amplitude of the beam gradually decreased. The displacement sensor of L5 was damaged during the measurement, and no valid data was obtained.

#### 3.3.3. Discussion

Combining the observed test phenomena and the test data, it could be seen that the failure process of the strengthened beam was similar to that of the control beam. Firstly, the concrete cracked, then the steel rebar yielded, finally the concrete in the compression area was crushed. The ECC cracked densely, but the CFRP grid didn’t break. The ECC and CFRP grid played a good role in dissipating energy. ECC significantly improved crack distribution. The high strength, high elastic modulus, and elastic properties of the CFRP grid made the residual deformation of the strengthened beam relatively small.

**Figure 12:**
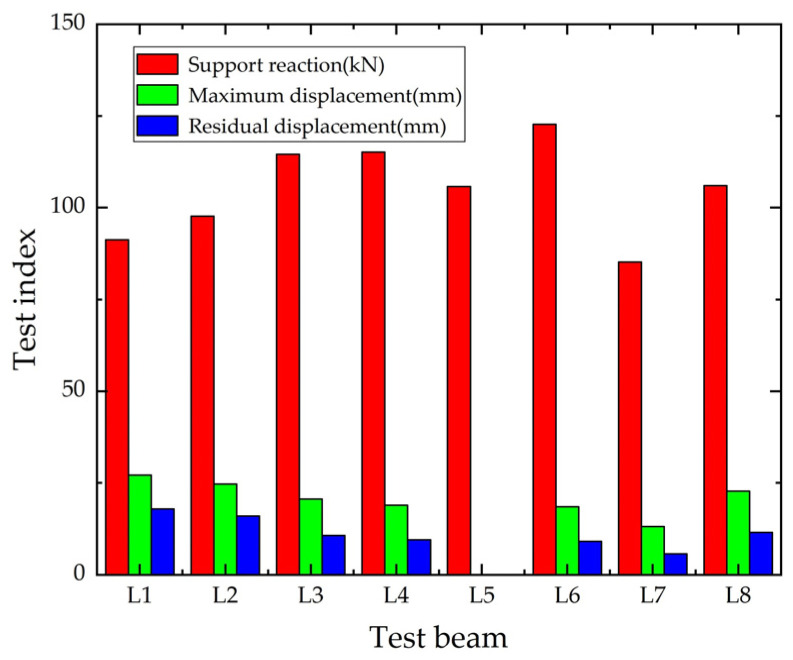
The maximum value of support reaction and mid-span displacement.

Figure 12 shows the maximum value of bearing reaction force, maximum value of mid-span displacement and mid-span residual displacement for each test beam. Compared with the control beam, the reaction force of RC beams strengthened with CFRP grid and ECC under impact load increased by 16.2%~34.5%, the maximum mid-span displacement decreased by 16.3%~31.6% and the mid-span residual displacement decreased by 36.02%~49.53%. Comparing L1 and L2, there was only a small increase in the support reaction force of L2, and the mid-span displacement was decreased, but the effect was limited, therefore, the improvement of the impact performance of RC beams by using only ECC was small. Comparing L2 and L3, the bearing reaction force of L3 was further increased and the maximum value of mid-span displacement and residual displacement were further reduced, so the CFRP grid reinforced ECC had a greater improvement on the impact resistance of RC beams. Comparing L3 and L4, the difference between the bearing reaction force and mid-span displacement between the two was very small, and no debonding/peeling of CFRP sheet was found during the tests. Therefore, in the case of no debonding/peeling of CFRP sheet, the influence of single-layer CFRP grid and CFRP sheet on the impact performance of RC beams was not much different. The displacement sensor of L5 was damaged and no displacement data was obtained. Comparing L3 and L5, the bearing reaction force of L5 was small, which was due to the construction difficulty of two-layer CFRP grid, resulting in uneven mixing of ECC, finally the bearing reaction force not reaching the expectation. Comparing L3 and L6, the bearing reaction force of L6 further increased, the mid-span displacement maximum and residual displacement decreased, so the increase of ECC thickness could improve the impact resistance of RC beam. Comparing L3 and L8, the bearing reaction force of L8 decreased and the maximum span displacement and residual displacement increase, so the 2% admixture of PE fiber had a more obvious effect on the improvement of beam impact resistance than the 1% admixture.

### 3.4. Time-History Curve of Strain

#### 3.4.1. Time-History Curve of CFRP Grid Strain

**Figure 13:**
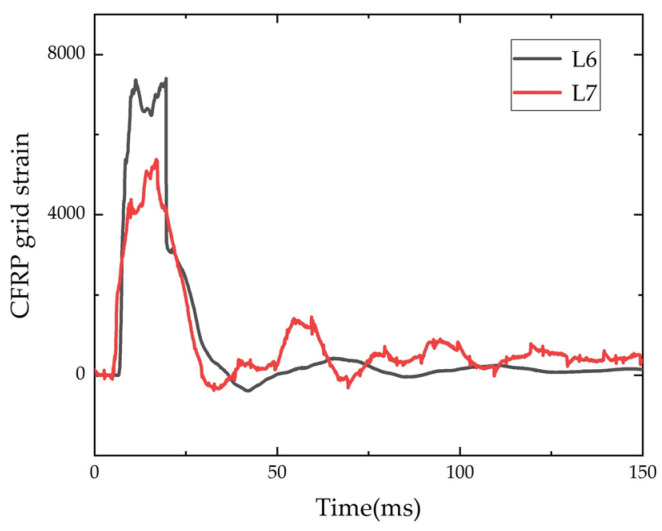
Time-history of CFRP grid strain.

The strain time-history curves of the CFRP grid of beam L6 and L7 are shown in Figure 13. The strain gauges of the remaining beams were damaged and no valid data could be measured. As can be seen from Figure 13, the CFRP grid strain increased rapidly to the peak under the impact, and then fell back and oscillated to 0. The peak value decreased during the oscillation process. The drop height of L7 was half of L6, and its grid strain was also much smaller than L6. Therefore, the higher the impact velocity, the strain on the CFRP grid in the mid-span of the strengthened beam would be higher.

#### 3.4.2. Time-History Curve of Carbon Fiber Sheet Strain

**Figure 14:**
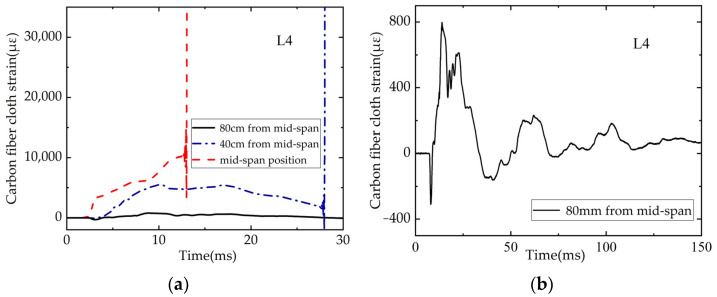
Time-history of CFRP sheet strain: (**a**) Strain comparison of three strain gauges; (**b**) Strain gauge at 80 cm from mid-span.

The strain time-history curve of carbon fiber sheet of L4 beam is shown in Figure 14. As can be seen from Figure 14a, under the impact of beam L4, the carbon fiber sheet strain gauge in the mid-span responded firstly, the strain increased rapidly and was out of the measurement range in 10 ms. The next response was the strain gauge at 40 cm from the mid-span. The strain increased first and then decreased and exceeds the range in 24 ms. The last response was the strain gauge at 80 cm from the mid-span as shown in Figure 14b, when the strain reached a peak and then continuously vibrated to decay to 0. Overall, under the impact, the strain of carbon fiber sheet at the bottom of the beam responded sequentially from the mid-span to both ends, and the magnitude and growth rate decreased sequentially from the mid-span to both ends.

#### 3.4.3. Time-History Curve of ECC Strain

**Figure 15:**
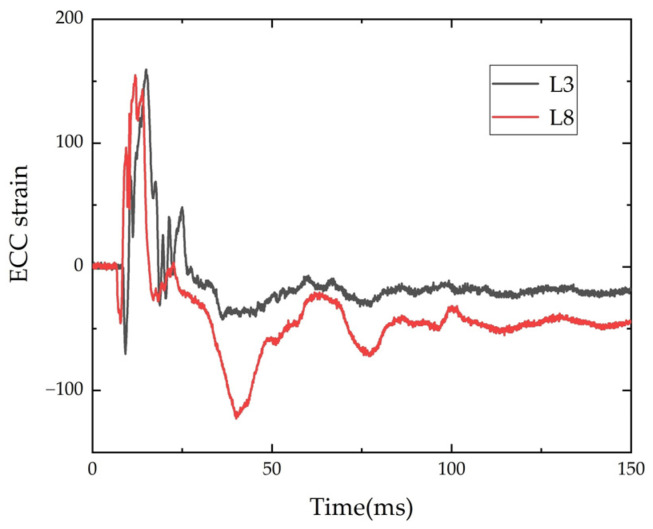
Time-history of ECC strain.

The strain time-history curves of the ECC of beam L3 and L8 are shown in Figure 15. It shows the ECC strain time-history curves of L3 and L8 at a distance of 80 cm from the mid-span. The rest of the ECC strain gauges were damaged and no valid data was measured. The strain gauge strain at 80 cm from the mid-span increased rapidly to the peak, then fell back and oscillated continuously to 0. The ECC produced fine cracks under the impact load, and the closer to the middle of the span, the more cracks there were, so the strain gauge in the mid-pan and 40 cm from the mid-span was severely damaged.

## 4. Finite Element Modeling

### 4.1. Establishment of Finite Element Models

**Figure 16:**
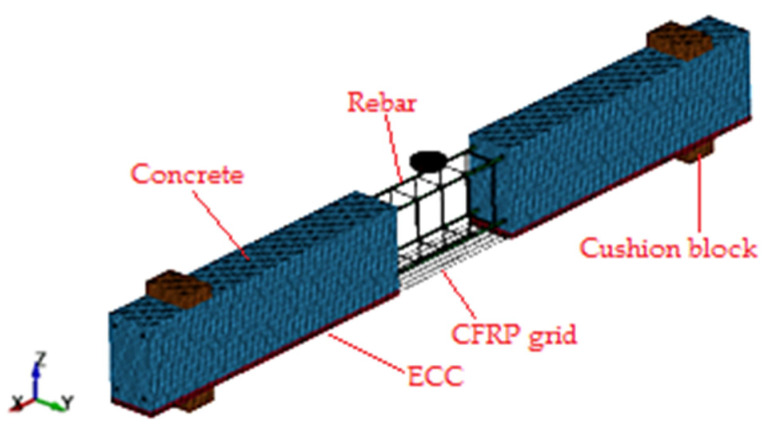
Finite element model of impact test.

For the problems of composite structures and impact [36,37], in order to obtain the strong nonlinear response details of the structure, as the verification and supplement of experimental data, explicit finite element method was usually used for numerical simulation. These studies demonstrated that finite element model and analysis procedure could predict the behavior of composite structures accurately. In this paper, LS-DYNA was used to build a finite element model of the RC beam as shown in Figure 16.

Concrete, ECC, drop hammer and pad were meshed with eight-node hexahedral solid element SOLID164, with single-point integration algorithm and Lagrangian mesh. The steel reinforcement and CFRP grid were meshed by two-node beam element BEAM161, with Hughes-Liu cell equation and 2 × 2 Gauss integration algorithm. The CFRP sheet was meshed by two-dimensional four-node quadrilateral shell element SHELL163, with Belytschko-Tsay algorithm and Gauss integration method.

The concrete material model was MAT_CSCM (MAT_159) concrete continuous cap model. The failure factor *ERODE* was 1.1 and the strain rate control parameter *IRATE* was 1. The ECC material model used the MAT_CONCRETE_DAMAGE_PLASTIC_MODEL (MAT_273) concrete plastic damage model. The hardening parameter *HP* was 0.5, the strain rate parameter *FC0* was 10^7^ Pa, and the strain rate parameter number *STRFLG* was 1. The compressive damage variable *EFC* was 0.001 as suggested by Grassl [38], the tensile damage type *TYPE* was type 1, and the maximum crack width $W_{f}$ was calculated from the tensile strength and fracture energy as follows.

(1) $$W_{f}=4.444\times \frac{G_{F}}{f_{t0}}$$

(2) $$G_{F}=73f_{c0}{}^{0.18}$$

where $G_{F}$ is the fracture energy, $f_{t0}$ is the tensile strength and $f_{c0}$ is the compressive strength. The remaining parameters were input from the actual measured values.

The MAT_PIECEWISE_ LINEAR_PLASTICITY (MAT_024) segmental linear elastic-plastic model was used for the reinforcement and grid material model. The strain rate parameter *SRC* was 40.4, and the strain rate parameter *SRP* was 5. The tensile test of the grid showed linear variation, and the ultimate strength was reached directly under the tensile force, and the shear modulus *ETAN* was set to 0. The drop hammer density was 8.35 × 10^5^ kg/m^3^, which was the calculated density obtained by dividing the total weight of the drop hammer and its upper steel beam by 200 kg and the volume of the drop hammer, and *CON1* and *CON2* were the constraints on the direction of the drop hammer’s translation and rotation. The MAT_ELASTIC (MAT_01) elastic material model was used for the mat material model. The density, modulus of elasticity and Poisson’s ratio were adopted from the data commonly used for steel. The carbon fiber sheet material model was adopted as MAT_ ENHANCED_COMPOSITE_DAMAGE (MAT_054) reinforced composite damage model. The values of literature [39] were used for the basic mechanical properties and the values of literature [40] were used for the failure parameters.

The thickness of ECC was 20 mm and the CFRP grid was located in the middle of ECC. The grid size was 10 mm in height direction. The spacing between carbon fiber strips in CFRP grid was 25 mm. The grid size was 12.5 mm in width direction and 10 mm in length direction. The keyword (* INITIAL_VELOCITY_GENERATION) was used to define the falling speed. The keywords (* CONTACT_ERODING_ SINGLE_SURFACE) and (* CONTACT_FORCE_ TRANSDUCER_PENALTY) were used to define the contact between the hammer and the beam.

### 4.2. Comparative Analysis of Numerical Simulation and Experimental Results

#### 4.2.1. Final Crack Pattern

**Figure 17:**
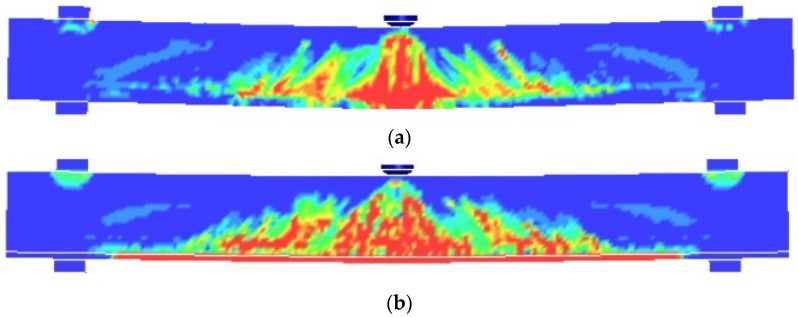
Cracking patterns of numerical simulation: (**a**) L1; (**b**) L3.

Taking the representative L1 and L3 as examples, the numerical simulation of the beam crack pattern is shown in Figure 17. The numerical simulation can reflect the crack distribution.

#### 4.2.2. Support Reaction Force Time-History Curve

**Figure 18:**
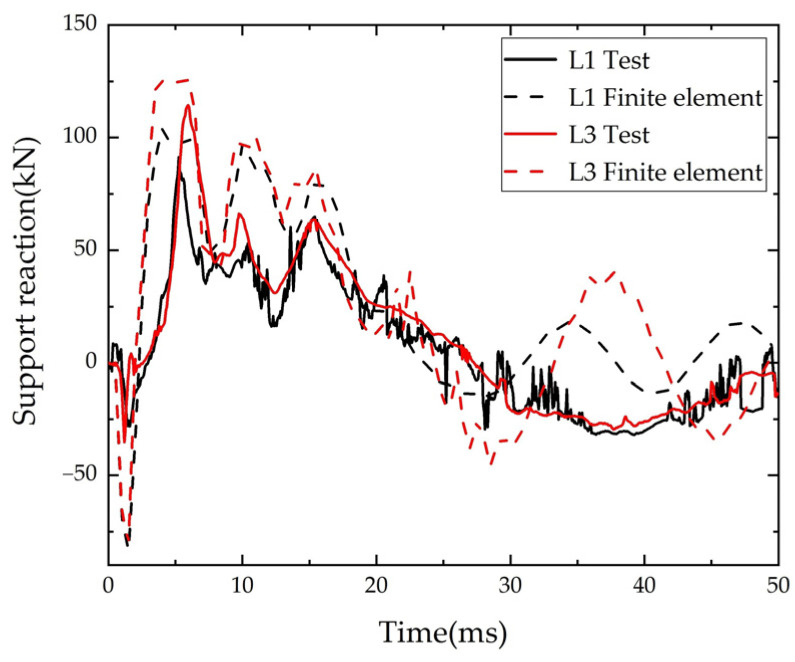
Comparison of time-history of reaction force.

The numerical simulation of the beam bearing reaction force and the comparison of the test results are shown in Figure 18. Although the peak value of bearing reaction force in the numerical simulation results was slightly larger than the test value, in general, the numerical simulation results were in good agreement with the test results.

#### 4.2.3. Mid-Span Displacement Time-History Curve

**Figure 19:**
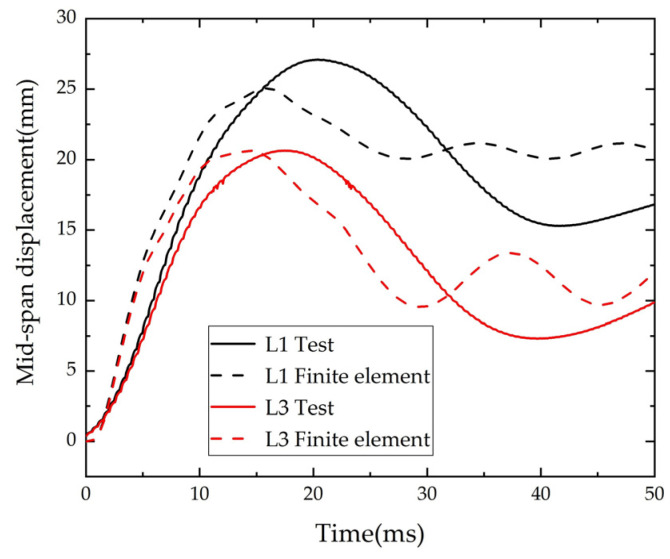
Comparison of time-history of displacement.

The comparison of the numerical simulation and test results of the beam mid-span displacement is shown in Figure 19. The maximum displacement and the time required to reach the peak displacement of the finite element model of mid-span displacement curve were in good agreement with the experimental values. The maximum displacement was very close to the test value, and the residual displacement after the peak value was slightly larger than the test value. This may be because the concrete stiffness recovery coefficient in the numerical simulation was based on the empirical default value of the program, which was slightly different from the actual situation.

### 4.3. Finite Element Parametric Analysis

The dynamic behavior of the CFRP grid reinforced ECC strengthened RC beams under impact loading was parametrically analyzed using the finite element modeling method. The analysis parameters included the thickness of the CFRP grid and the reinforcement rate of the longitudinal tensile reinforcement, and the remaining conditions of the simulated beam are the same as those of the test beam L3.

#### 4.3.1. Thickness of CFRP Grid

**Figure 20:**
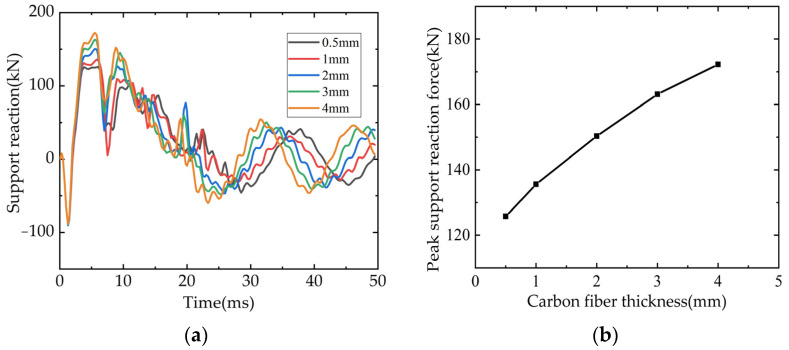

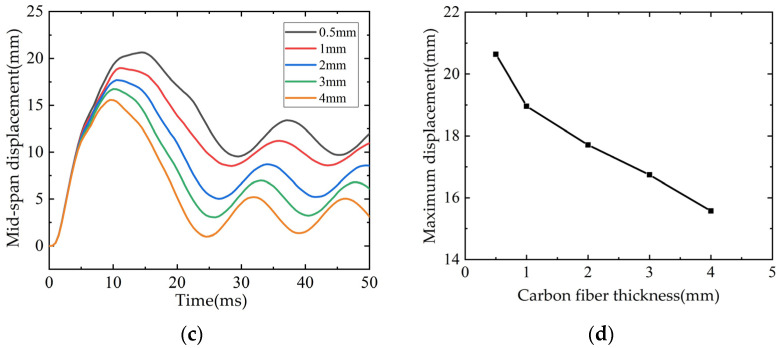
Influence of CFRP thickness on beams: (**a**) Time-history curve of support reaction; (**b**) Bearing reaction peak/carbon fiber thickness curve; (**c**) Time-history curve of mid-span displacement; (**d**) Maximum displacement in mid-span/carbon fiber thickness curve.

The thickness of carbon fiber in the CFRP grid of L3 beam was 0.5 mm, and the thickness of carbon fiber was set to 1 mm, 2 mm, 3 mm and 4 mm, respectively. The time curves of bearing reaction force and mid-span displacement and the curves of thickness were shown in Figure 20. From Figure 20a,b, it can be seen that with the increase of carbon fiber thickness, the maximum value of beam support reaction force gradually increases. From Figure 20c,d, it can be seen that the maximum value of the mid-span displacement and the residual value of the beam gradually decrease with the increase of the carbon fiber thickness, and the time required reaching the maximum displacement also gradually decreases.

#### 4.3.2. Longitudinal Reinforcement Rate of Tensile Steel

**Figure 21:**
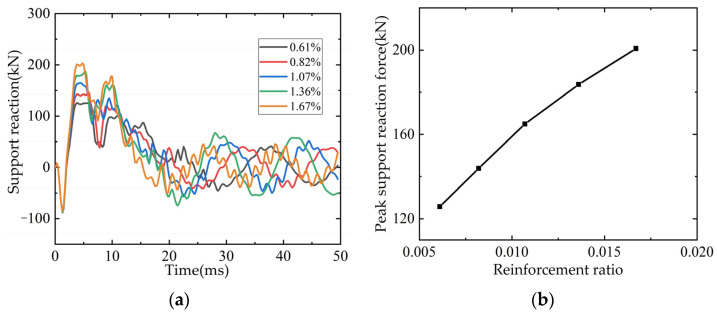

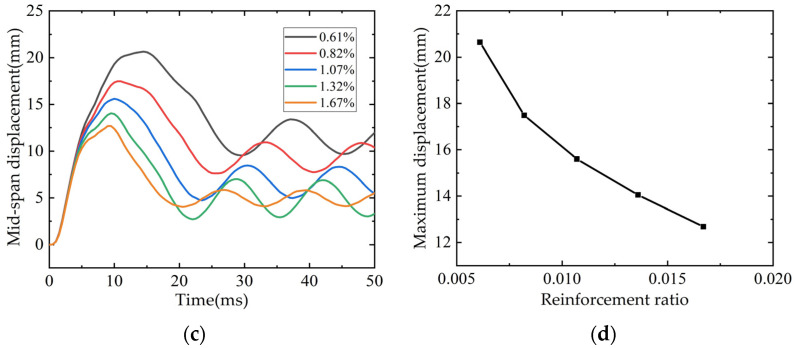
Influence of reinforcement ratio on beams: (**a**) Time-history curve of support reaction; (**b**) Bearing reaction peak/reinforcement ratio curve; (**c**) Time-history curve of mid-span displacement; (**d**) Maximum displacement in mid-span/reinforcement ratio curve.

The longitudinal tensile reinforcement diameter of L3 beam was 12 mm, and the reinforcement rate was 0.61%. Considering that the reinforcement rate of longitudinal tensile reinforcement should not exceed 2.5%, the corresponding reinforcement rates were 0.82%, 1.07%, 1.36% and 1.67% after setting the diameters of reinforcement to 14 mm, 16 mm, 18 mm and 20 mm, respectively. The time curves of bearing reaction force and mid-span displacement and the relationship curves with reinforcement ratio are shown in Figure 21. From Figure 21a,b, it can be seen that with the increase of longitudinal tensile reinforcement, the bearing reaction force increases. From Figure 21c,d, it can be seen that the maximum mid-span displacement decreases as the reinforcement ratio of longitudinal tensile reinforcement increases.

## 5. Simplified TDOF Model Analysis of Strengthened Beam

### 5.1. Parameter and Solution of Simplified TDOF Model

**Figure 22:**
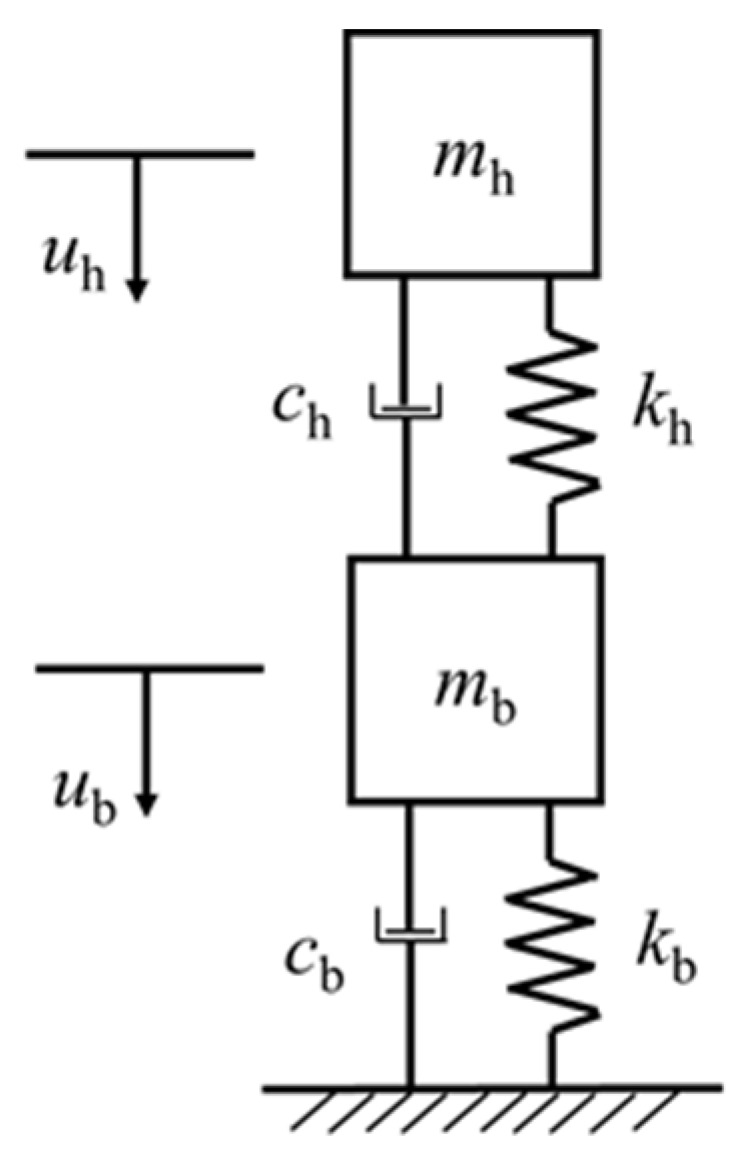
Simplified TDOF model.

The simplified TDOF model is shown in Figure 22. $m_{h}$ is the mass of the falling hammer, $m_{b}$ is the equivalent mass of the beam, $k_{h}$ and $c_{h}$ are the contact stiffness and contact damping of the falling hammer and the beam, $k_{b}$ and $c_{b}$ are the stiffness and damping of the beam. The response of the beam under the impact of the falling hammer is simulated by applying the initial velocity and initial acceleration to $m_{h}$.

#### 5.1.1. Mass $m_{b}$ Mechanical Model

The equivalent mass $m_{b}$ of the beam is calculated according to the principle of equal kinetic energy before and after equivalence. The kinetic energy $I_{k0}$ of the beam with uniform mass before equivalence and the kinetic energy $I_{\mathrm{ke}}$ of the mass after equivalence are shown in the following Equation:

(3) $$I_{k0}=\frac{1}{2}\rho A\int_{0}^{l}{\left[v_{m}\cdot \psi (x)\right]}^{2}dx$$

(4) $$I_{\mathrm{ke}}=\frac{1}{2}m_{b}{}^{2}v_{m}{}^{2}$$

where *ρ* is the beam density, *A* is the beam cross-sectional area, and ψ(x) is the beam deflection shape function. Let the two equations be equal, the expression of $m_{b}$ can be obtained as follows:

(5) $$m_{b}=\rho A\int_{0}^{l}{\left[\psi (x)\right]}^{2}dx$$

**Figure 23:**
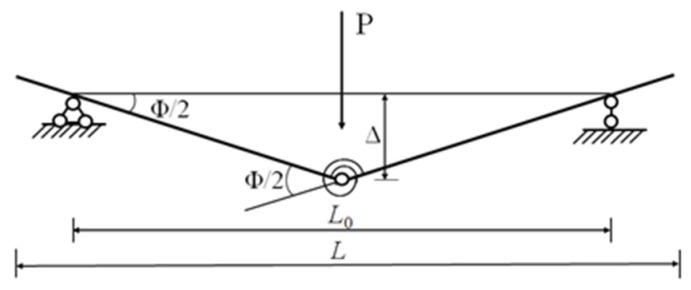
Simplified beam model.

Researchers usually use one of triangle, sinusoidal half-wave or deflection curve shape under static load to describe the deflection shape function of RC beams subjected to impact action in the span. In the test, the plastic deformation of the beam is more obvious, and the plastic deformation is mainly concentrated in the middle of span. Therefore, the beam deflection curve is assumed triangular in this paper, and the simplified model of the beam is shown in Figure 23.

Where *L* and $L_{0}$ are the total length and net span of the beam, respectively, *Φ* is the angle of rotation at the plastic hinge, and Δ is the vertical displacement in the beam mid-span as shown in the following equation:

(6) $$m_{b}=\frac{1}{3}\rho A\left[L_{0}+{\left(L-L_{0}\right)}^{3}/L_{0}{}^{2}\right]$$

#### 5.1.2. Spring $k_{b}$ Mechanical Model

**Figure 24:**
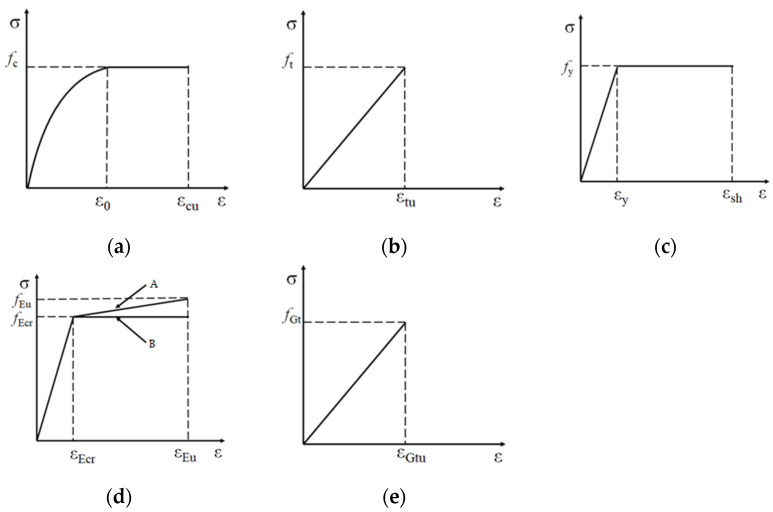
Material constitutive model: (**a**) Concrete under compression; (**b**) Concrete under tension; (**c**) Reinforcement under tension; (**d**) ECC under tension; (**e**) CFRP grid under tension.

From the beam design parameters and material constitutive information, the beam cross-sectional moment-curvature relationship can be obtained, then the force-deflection relationship can be obtained, and finally, the beam stiffness $k_{b}$ can be obtained. The material constitutive model involved in the strengthened beam is shown in Figure 24.

The compression constitutive equation of concrete is:

(7) $$\sigma_{c}=\left\{ \begin{array}{ll} f_{c}\left[1-{\left(1-\frac{\varepsilon }{\varepsilon_{0}}\right)}^{2}\right] & \varepsilon \leq \varepsilon_{0}\\ f_{c} & \varepsilon >\varepsilon_{0}\; \end{array}\right.$$

The tensile constitutive equation of concrete is:

(8) $$f_{t}=0.395f_{c}^{0.55}$$

The tensile constitutive equation of reinforcement is:

(9) $$\sigma_{s}=\left\{ \begin{array}{ll} E_{s}\varepsilon & \varepsilon \leq \varepsilon_{y}\\ f_{y} & \varepsilon >\varepsilon_{y}\; \end{array}\right.$$

In Figure 24d the path ‘A’ can accurately reflect the ECC tensile principal structure relationship, but to simplify the calculation, path ‘B’ is used. The constitutive equation is:

(10) $$\sigma_{E}=\left\{ \begin{array}{ll} E_{E}\varepsilon & \varepsilon \leq \varepsilon_{\mathrm{Ecr}}\\ f_{\mathrm{Ecr}} & \varepsilon >\varepsilon_{\mathrm{Ecr}}\; \end{array}\right.$$

The constitutive equation of CFRP grid is:

(11) $$\sigma_{G}=E_{G}\varepsilon_{G}$$

where $E_{c}$, $E_{s}$, $E_{E}$ and $E_{G}$ are the modulus of elasticity of concrete, reinforcement, ECC and CFRP grid, respectively, $f_{c}$ and $f_{t}$ are the compressive and tensile strengths of concrete,$f_{y}$ is the yield strength of reinforcement and $f_{Ecr}$ is the cracking strength of ECC.

The strain rate amplification effect of the beam strength under impact should be considered in the simplified model. The strain rate effect of concrete strength is adopted from the CEB-FIP model specification (2010), and the formulae for the dynamic amplification coefficients of concrete compressive and tensile strengths are given below:

(12) $$f_{c,\mathrm{imp}}/f_{c}=\left\{ \begin{array}{ll} {\left({\dot{\varepsilon }}_{c}/{\dot{\varepsilon }}_{c0}\right)}^{0.014} & \left|{\dot{\varepsilon }}_{c}\right|\leq 30s^{-1}\\ 0.012{\left({\dot{\varepsilon }}_{c}/{\dot{\varepsilon }}_{c0}\right)}^{1/3} & \left|{\dot{\varepsilon }}_{c}\right|>30s^{-1}\; \end{array}\right.$$

(13) $$f_{\mathrm{ct},\mathrm{imp}}/f_{t}=\left\{ \begin{array}{ll} {\left({\dot{\varepsilon }}_{\mathrm{ct}}/{\dot{\varepsilon }}_{\mathrm{ct}0}\right)}^{0.018} & \left|{\dot{\varepsilon }}_{\mathrm{ct}}\right|\leq 10s^{-1}\\ 0.0062{\left({\dot{\varepsilon }}_{\mathrm{ct}}/{\dot{\varepsilon }}_{\mathrm{ct}0}\right)}^{1/3} & \left|{\dot{\varepsilon }}_{\mathrm{ct}}\right|>10s^{-1}\; \end{array}\right.$$

where $f_{c,imp}$ and $f_{ct,imp}$ are the compressive and tensile strength of concrete considering the amplification effect of strain rate, respectively. ${\dot{\varepsilon }}_{c0}$ is taken as −30 × 10^−6^ s^−1^, and ${\dot{\varepsilon }}_{\mathrm{ct}0}$ is taken as −10^−6^ s^−1^. According to Guo [41], the strain rate of concrete is taken as 2 s^−1^ in the impact test of beam drop hammer, and the dynamic amplification coefficients of compressive and tensile strength of concrete are 1.17 to 1.3. The amplification coefficient of yield strength of rebar is taken as 1.20 recommended by scholars after high-speed tensile test of HRB400 rebar [42]. There are few studies on the dynamic amplification coefficients of ECC and CFRP grid, this paper refers to the dynamic amplification coefficient of tensile strength of concrete and rebar.

**Figure 25:**
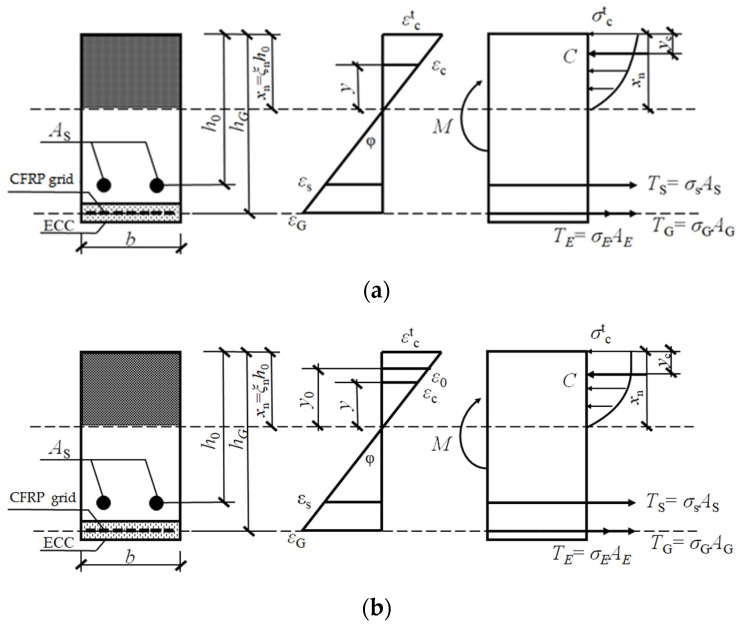
Calculation diagram of strengthened beam section: (**a**) Cross-section calculation diagram ($\varepsilon_{c}^{t}\leq \varepsilon_{0}$); (**b**) Cross-section calculation diagram ($\varepsilon_{c}^{t}>\varepsilon_{0}$ ).

According to the concrete strain $\varepsilon_{c}^{t}$ and $\varepsilon_{0}$(0.002) at the compressed edge, the sketch of the section calculation can be divided into two cases as shown in Figure 25.

Where *C* is the combined concrete force in the compression zone, $T_{S}$ is the reinforcement tension, $T_{E}$ is the ECC tension, and $T_{G}$ is the CFRP grid tension. Since the ECC strengthening layer is thin, it is assumed that the strain at the ECC strengthening layer is equal to the strain at the CFRP grid. For both cases, the bending moment values at the corresponding section strains can be calculated by the combined force and moment balance equations (Equations (14) and (15)).

(14) $$C=T_{S}+T_{E}+T_{G}$$

(15) $$M=T_{s}\cdot \left(h_{0}-y_{c}\right)+(T_{E}+T_{G})\cdot (h_{G}-y_{c})$$

The first stage ends with ECC cracking. Since the cracking strain of ECC is greater than that of concrete and the ECC is thinner, the concrete is already cracked when the ECC cracks and the concrete tension in the tensile zone can be ignored when calculating the ECC cracking load. When ECC cracking, let $\varepsilon_{E}=\varepsilon_{Ecr}$, according to the Equations (14) and (15), we can acquire ECC cracking moment $M_{cr}$.

The second stage ends with the yielding of the longitudinal tensile rebar. When the rebar yields, let $\varepsilon_{s}=\varepsilon_{y}$, according to Equations (14) and (15) can be obtained from the rebar yield moment $M_{y}$.

The third stage ends with the crushing of concrete in the compression zone. When the beam is damaged, let $\varepsilon_{c}^{t}=\varepsilon_{cu}$, and according to Equations (14) and (15), we can acquire the damage bending moment $M_{u}$.

**Figure 26:**
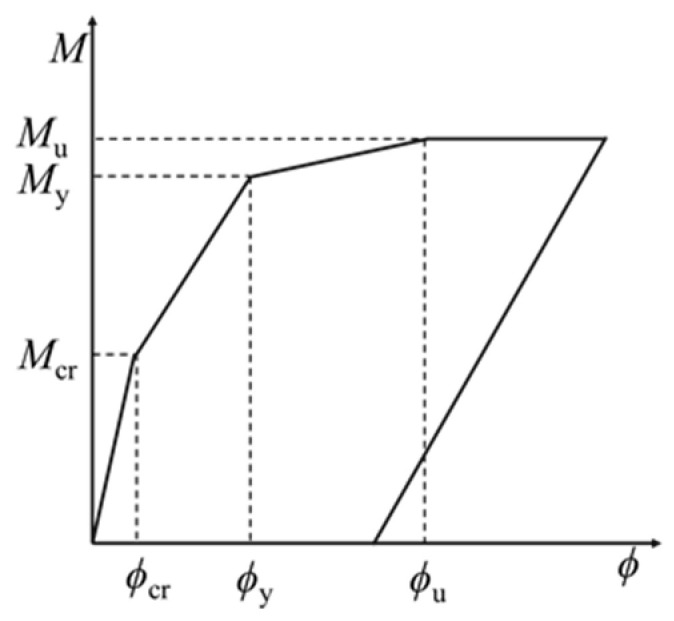
Moment-Curvature diagram.

The resulting bending moment *M*-curvature *ϕ* relationship for the strengthened beam is shown in Figure 26. The mid-span turning angle $\Phi =\varphi L_{p}$, $L_{p}$ is the length of the plastic section in the mid-span, which can be calculated by the formula [34]:

(16) $$L_{P}=h_{0}+0.05L_{0}$$

**Figure 27:**
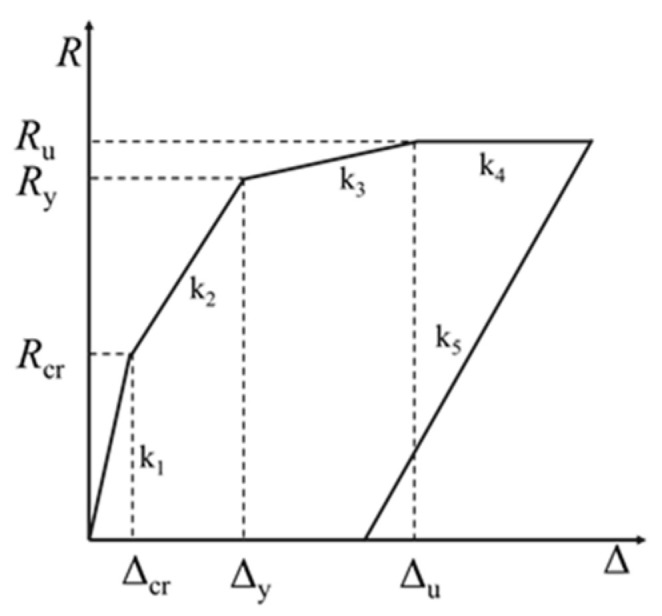
Restoring force-deflection diagram.

It can be seen from Figure 23 that the mid-span deflection of the beam $\Delta =\Phi L_{0}/4$, and the restoring force of the strengthened beam $R=4M/L_{0}$. The relationship between the restoring force of the strengthened beam *R* and the mid-span deflection Δ is shown in Figure 27. $k_{1}$, $k_{2}$ and $k_{3}$ represent the stiffness of the strengthened beam before ECC cracking, before the yield of the rebar and before the crushing of the concrete in the compression zone, respectively, $k_{4}$ represents the stiffness of the strengthened beam after crushing of the concrete in the compression zone, and $k_{5}$ represents the stiffness of the unloading stage. The $k_{2}$ recommended by Zhao [34] is adopted.

#### 5.1.3. Damping $c_{b}$ Mechanical Model

The damping ratio $\xi_{b}$ of the strengthened beam is taken as 0.05 for reinforced concrete structures in structural design, and the damping $c_{b}$ of the strengthened beam can be solved by the following equation:

(17) $$c_{b}=0.1\sqrt{k_{b}m_{b}}$$

#### 5.1.4. Spring $k_{h}$ Mechanical Model

The contact stiffness $k_{h}$ of the falling hammer and the strengthened beam is calculated according to the Hertz contact theory [43]. The surface of the falling hammer is a spherical crown, the contact with the beam can be simplified to a spherical-plane contact, and the following equation can be obtained according to the Hertz contact theory:

(18) $$\delta^{3}=\frac{9}{16}\cdot \frac{1}{R_{0}}{\left(\frac{1-\mu_{h}{}^{2}}{E_{h}}+\frac{1-\mu_{b}{}^{2}}{E_{b}}\right)}^{2}\cdot P^{2}$$

where *δ* is the deformation variable, *P* is the loading external force, $R_{0}$ is the radius of the falling hammer, $\mu_{h}$ and $\mu_{b}$ are the Poisson’s ratio of the hammer head and the beam, $E_{h}$ and $E_{b}$ are the modulus of elasticity of the hammer head and the beam. The contact stiffness $E_{h}$ is calculated to be 2.5 × 10^8^ N/m.

#### 5.1.5. Damping $c_{h}$ Mechanical Model

The contact damping $c_{h}$ is adopted as suggested by Fujikake [44], taking half of the critical damping as shown in the following equation:

(19) $$c_{h}=\sqrt{\frac{m_{h}m_{b}}{m_{h}+m_{b}}k_{h}}$$

#### 5.1.6. Computational Solution

After calculating each parameter, it can be substituted into the following motion equations to solve:

(20) $$\left[\begin{array}{cc} m_{h} & 0\\ 0 & m_{b} \end{array}\right]\left\{\begin{array}{c} {\ddot{u}}_{h}\\ {\ddot{u}}_{b} \end{array}\right\}+\left[\begin{array}{cc} c_{h} & -c_{h}\\ -c_{h} & c_{h}+c_{b} \end{array}\right]\left\{\begin{array}{c} {\dot{u}}_{h}\\ {\dot{u}}_{b} \end{array}\right\}+\left[\begin{array}{cc} k_{h} & -k_{h}\\ -k_{h} & k_{h}+k_{b} \end{array}\right]\left\{\begin{array}{c} u_{h}\\ u_{b} \end{array}\right\}=\left\{\begin{array}{c} m_{h}g\\ 0 \end{array}\right\}$$

To ensure accuracy, the Runge-Kutta-Fehlberg (RKF45) method [45] was used to obtain the response of the structure during the impact by inputting the parameters of different strengthened beams.

### 5.2. Comparison Analysis of Simplified Model and Experimental Results

**Figure 28:**
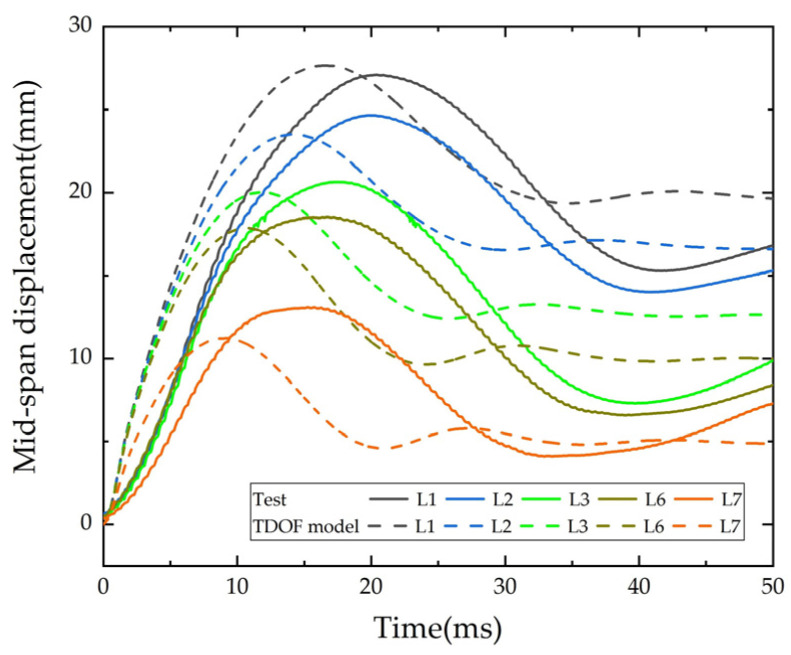
Comparison of time-history of displacement.

The input data for the simplified model calculation are shown in Table 7, the calculated time-history curves of mid-span deflection of L1, L2, L3, L6 and L7 under impact are shown in Figure 28.

By comparing the simplified model with the experimental results, the average error of the mid-span maximum displacement is 4.7% with a standard deviation of 0.0589; the average error of the mid-span residual displacement is 4.5% with a standard deviation of 0.109. The errors of the maximum displacement and the residual displacement in the mid-span are very low, which indicates that the simplified TDOF model in this paper can simulate the key characteristics of impact response of the strengthened beam effectively. However, some data are not obtained in this test, so the recommended values in the relevant literature are adopted, such as $k_{2}$ (the beam stiffness before rebar yielding) and the strength amplification coefficient of each material, which may be the reason for the incomplete consistency between the simulation results and the test results.

### 5.3. Parameter Analysis with Simplified Model

Using the simplified model, the dynamic behavior of CFRP grid reinforced ECC strengthened RC beams under impact loading was analyzed parametrically. The analysis parameters include the shear span ratio of the beam, the impact velocity of the falling hammer and the mass of the falling hammer, and the rest of the conditions are the same as those of the test beam L3.

#### 5.3.1. Shear Span Ratio

**Figure 29:**
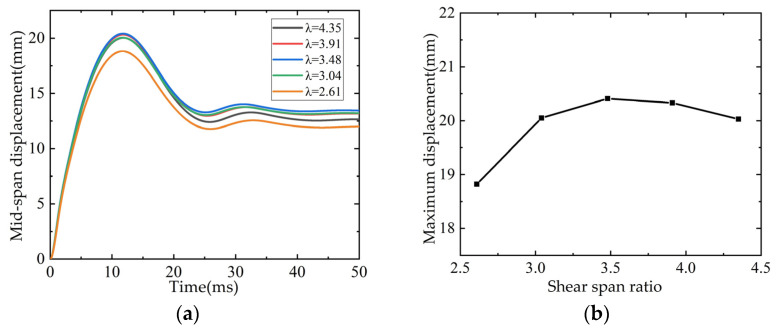
Influence of shear span ratio on mid span displacement: (**a**) Time-history curve of mid-span displacement; (**b**) Maximum displacement in mid-span/shear span ratio curve.

The net span of beam L3 is 2 m, and the shear span ratio (4.35) is the ratio of the distance from the point of concentrated load to the edge of the support to the effective height of the section. The net span of the beam is set to 1.8 m, 1.6 m, 1.4 m and 1.2 m, and the corresponding shear span ratios (λ) are 3.91, 3.48, 3.04 and 2.61. The time curve of mid-span displacement and the relationship curve with shear span ratio are shown in Figure 29.

#### 5.3.2. Drop Hammer Impact Velocity

**Figure 30:**
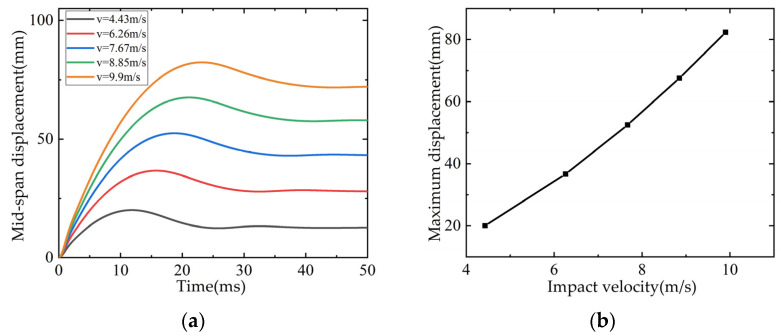
Influence of impact velocity on mid-span displacement: (**a**) Time-history curve of mid-span displacement; (**b**) Maximum displacement in mid-span/shear span ratio curve.

L3 beam drop hammer mass 200 kg, drop height 1 m, impact velocity 4.43 m/s. When the influence of speed is considered in the parameter analysis, the weight of the drop weight is kept unchanged, and the drop heights are set to 2 m, 3 m, 4 m and 5 m, respectively, and the corresponding impact speeds are 6.26 m/s, 7.67 m/s, 8.85 m/s and 9.9 m/s. The time-history curve of the mid-span displacement and the relationship curve with the drop weight velocity are shown in Figure 30. It can be seen from the figure that with the increase of the drop weight velocity, the maximum value of the mid-span displacement increases monotonically, and the growth rate increases gradually.

#### 5.3.3. Drop Weight

**Figure 31:**
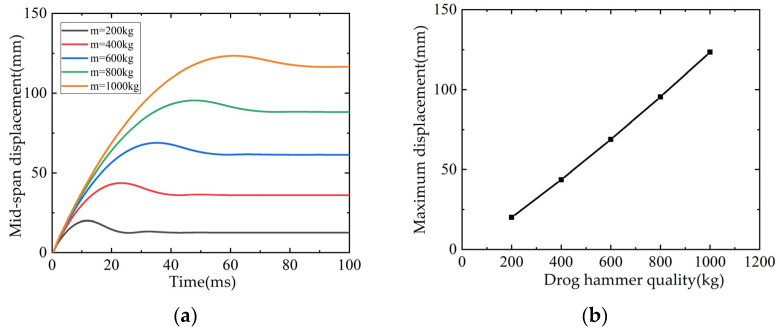
Influence of falling weight on mid span displacement: (**a**) Time-history curve of mid-span displacement; (**b**) Maximum displacement in mid-span/shear span ratio curve.

The weight of the drop weight of the L3 beam is 200 kg, the drop height is 1 m, and the impact speed is 4.43 m/s. When the parameter analysis considers the influence of the mass, keep the drop weight speed unchanged, and the drop weight is set to 400 kg, 600 kg, 800 kg and 1000 kg, respectively. The displacement time-history curve and the relationship curve with mass are shown in Figure 31. It can be seen from the figure that with the increase of the mass of the drop weight, the maximum mid-span displacement increases approximately linearly and monotonically.

## 6. Conclusions

In this paper, the impact test of RC beams strengthened with ECC and CFRP grid was carried out, then the finite element model was used to simulate the impact test, finally a simplified TDOF model was proposed that can be used to calculate the mid-span deflection time curve. The following conclusions can be drawn:

- When the ECC and CFRP grid were used, the crack development was inhibited after the concrete cracked in the tensile area, and the tensile force was conducted to the uncracked concrete area, avoiding the brittle damage of RC beams with one crack to the end. CFRP grid reinforced ECC strengthened layer could improve the impact resistance of RC beams. The support reaction force was improved by 16.2%~34.5%, maximum mid-span displacement was reduced by 16.3%~31.6%, and mid-span residual displacement was reduced by 36.02%~49.53%. Too many layers of CFRP grid might lead to uneven ECC pouring and reduce the strengthening effect. The increase of ECC thickness could increase the peak of support reaction force and reduce the mid-span displacement. The 2% admixture of PE fibers in the ECC layer had a more significant effect on the lifting of the beam support reaction force and the reduction of the mid-span displacement than the 1% admixture. CFRP grid and ECC strengthened beams were close to the strengthening effect in the case where the nominal cross-sectional area of carbon fiber was much smaller than that of carbon fiber sheet and ECC strengthened beams.
- The finite element model could effectively predict the crack distribution of CFRP grid reinforced ECC strengthened RC beams. The parameters analysis with the finite element model revealed that the peak bearing reaction force increased with the increase of CFRP grid thickness, and the maximum displacement in the mid-span decreased with the increase of CFRP grid thickness. The peak bearing reaction force increased with the increase of longitudinal reinforcement rate, and the maximum displacement in the mid-span decreased with the increase of longitudinal reinforcement rate.
- The simplified TDOF model could simulate the impact response of the reinforced beam accurately. The parameter analysis with the simplified TDOF model showed that the maximum mid-span displacement of the beam increased first and then decreased slightly with the increase of the shear span ratio. The maximum mid-span displacement increased monotonically with the increase of the falling hammer speed and the growth rate increased gradually. With the increase of the falling hammer quality, the maximum mid-span displacement increased linearly and monotonically.

Aiming at the influence of various parameters concerned in engineering applications on the impact performance of FRP grid reinforced ECC strengthening beams, impact tests were firstly carried out in this paper, then supplementary research was carried out with finite element analysis and simplified theoretical calculation model. The research results in this paper can be used for reference by researchers and engineering technicians when carrying out related research and applications. However, it is worth noting and needing further research in the following aspects. The construction method of CFRP grid reinforced ECC needs to be improved. The construction of multi-layer CFRP grid is difficult and the fibers in ECC are not easily mixed uniformly, which may seriously affect the strengthening effect. The simplified TDOF model also needs to be further refined. Therefore, the conclusions are only applicable to the cases studied in this work.

## Figures and Tables

**Table 1:** Mechanical property of rebar.

Rebar Diameter (mm)	Model	Yield Strength (MPa)	Ultimate Strength (MPa)	Elastic Modulus (GPa)
12	HRB400	452	601	208
6	HPB300	404	558	201

**Table 2:** Mechanical property of ECC.

Compressive Strength (MPa)	Flexural Strength (MPa)	Cracking Strength (MPa)	Tensile Strength (MPa)
55.16	16.45	5.05	6.66

**Table 3:** Mechanical property of carbon fiber sheet.

Tensile Strength (MPa)	Elastic Modulus (GPa)	Poisson Ratio	Nominal Thickness (m)
3513	240	0.307	0.000167

**Table 4:** Mechanical property of FRP grid.

Material	Tensile Strength (MPa)	Elastic Modulus (GPa)	Poisson Ratio	Nominal Thickness (m)	Nominal Cross-Sectional Area (m^2^)
CFRP	4900	230	0.307	0.0005	0.00009
GFRP	3430	73	0.22	0.0003	0.00005

**Table 5:** Parameters of test beam.

Number	Strengthening Method	Fiber Content	ECC Thickness(mm)	Impact Height (m)	Grid Layers
L1	—	0	0	1	0
L2	ECC	2%	20	1	0
L3	grid + ECC	2%	20	1	1
L4	carbon fiber sheet + ECC	2%	20	1	0
L5	grid + ECC	2%	20	1	2
L6	grid + ECC	2%	40	1	1
L7	grid + ECC	2%	20	0.5	1
L8	grid + ECC	1%	20	1	1

**Table 6:** The development of cracks.

	Crack Pattern	2–5 ms	10–15 ms	15–20 ms	20–25 ms
L1	Main Crack	2/5 h	3/5 h	4/5 h	-
	Horizontal cracks	-	-	Appear	-
L2	Main Crack	1/2 h	3/5 h	-	7/10 h
	Horizontal cracks	-	Appear	-	-
L3	Main Crack	2/5 h	7/10 h	4/5 h	-
	Horizontal cracks	-	Appear	-	-
L4	Main Crack	1/2 h	-	3/5 h	-
	Horizontal cracks	-	Appear	-	-

**Table 7:** Input data of Simplify model.

Number	$m_{b}$	$c_{h}$	$c_{b}$	$k_{1}$	$k_{2}$	$k_{3}$	${△}_{\mathrm{cr}}$	${△}_{y}$	${△}_{u}$	*v*
L1	63.0	1.09	3.02	7.85	1.44	2.23	2.49	2.34	12.6	4.43
L2	67.4	1.12	3.73	3.46	2.06	2.91	5.90	2.43	10.9	4.43
L3	67.4	1.12	3.83	3.57	2.18	21.1	5.95	2.47	8.78	4.43
L5	67.4	1.12	3.93	3.68	2.29	36.9	6.00	2.50	7.75	4.43
L6	71.9	1.15	3.94	4.85	2.16	22.7	6.09	2.56	8.00	4.43
L7	67.4	1.12	3.83	3.57	2.18	21.1	5.95	2.47	8.78	3.13

## Data Availability

Data is contained within the article.
